# A case report of mesenteric mucinous cystoadenoma with review of the literature

**DOI:** 10.1186/1471-230X-10-105

**Published:** 2010-09-14

**Authors:** Alessandro Del Gobbo, Manuela Bimbatti, Stefano Ferrero

**Affiliations:** 1 Department of Medicine, Surgery and Dentistry, Division of Pathology, AO S. Paolo e Fondazione IRCCS Ospedale Maggiore Policlinico, Mangiagalli and Regina Elena, University of Milan Medical School, Milan, Italy

## Abstract

**Background:**

Few cases of primary retroperitoneal mucinous cystoadenoma, a rare benign tumor, have been reported in the literature so far. The pathogenesis of this tumour is not completely understood yet.

Our case is particularly significant since the localization in the mesentery has been described only once before in the literature. Unless biologically benign, this tumour can cause relevant clinical symptoms related to the size and site (compression or obstruction of organs).

**Case presentation:**

We describe the case of a 52-years old woman who had presented with abdominal pain and underwent surgery in order to remove a palpable lump in the mesentery with histological diagnosis of primary mucinous cystoadenoma. The patient was followed-up for two years with no evidence of recurrence.

**Conclusions:**

Mucinous cystoadenoma is more frequent in women, particularly when there is history of one or more pregnancies. A complete preoperative study with abdominal and pelvic tomographic images and an accurate physical examination are essentials for the management of the patient. Surgical resection is the only way to treat mucinous cystoadenomas, and to have the histological confirmation that the removed mass is a benign tumor.

## Background

A few cases of retroperitoneal mucinous cystoadenoma (RMC), a rare benign tumor, are reported in the literature [1-6]. This is the second case reported in the literature of mesenteric localization of mucinous cystoadenoma to our best knowledge [1].

Cystoadenoma causes symptoms linked to its size and site as do many masses localized in the abdominal cavity or retroperitoneum, where frequently the adjacent structures like blood vessels, ureters, small and/or large bowel tracts can be compressed or obstructed. The symptom more frequently described in these cases is the abdominal pain, sometimes exacerbated by the compression practiced on the mass during medical examination.

## Case Presentation

A 52 year-old woman came to Hospital complaining a vague feeling of heaviness in right lower quadrant in the last few months. The woman had had two pregnancies and the anamnesis did not put into evidence any pathologically relevant note.

The medical examination revealed a deep palpable mass in the abdomen; blood tests were normal, except for the modest raising of the ALT equal to 65 U/Ls (normal range 7-41), CEA equal to 13.58 (normal range: 0.0-4.6), and of the CA 19.9 equal to 43.55 (normal range 0.0-37).

Transvaginal ultrasound features of the mass showed an egg-shaped cystic mass of nearly 14 cm in diameter, uniloculated, with liquid content, and thickened walls with foci of calcification. Colonoscopy revealed compression of terminal ileum and right colon with normal mucosa. The lump was deeply located in the right lower quadrant, in anatomical relationship with ileum, and causing the compression of the right ureter; the ovaries and the female reproductive system were normal. The patient did not undergo abdominal or pelvic computed tomography, since both of them were considered to be unnecessary by the surgeon.

The surgical intervention allowed the removal of the retroperitoneal mass with terminal ileum, right colon, right ovarian vessels and right ureter, which were embedded in the mass. The continuity of the right ureter was guaranteed with a "double J" prosthesis uretero-ureteroanastomosis. Intraoperative examination excluded neoplastic involvement of the resected right ureter.

**Figure 1:**
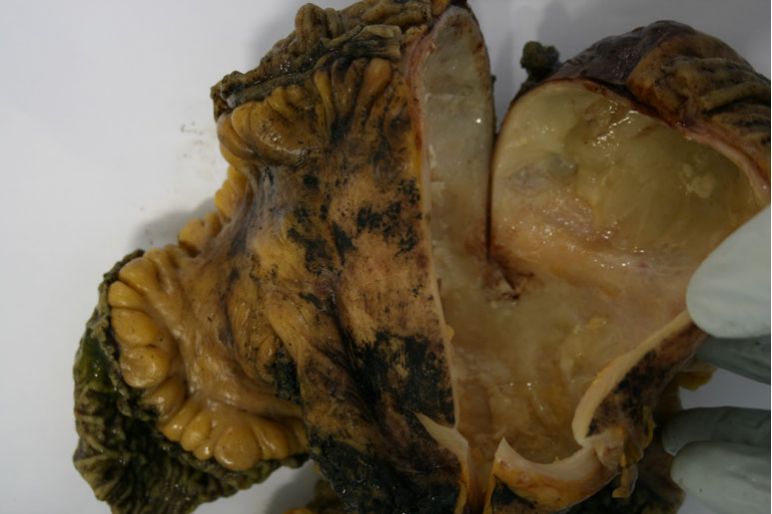
**Gross view of the specimen**.

A mass of 10,5 cm of major diameter was entirely embedded in the mesentery, from which it was easily removed except for an area of firm adhesion to surrounding connective tissue corresponding to terminal ileum, the external surface was smooth and the cut-up showed a cystic lesion with smooth internal surface, containing mucinous whitish fluid (fig. 1).

The wall and the mucosa of both small and large bowel and the right ureter appeared grossly uninvolved by the lesion.

The most common differential diagnoses include other neoplastic lesions such as cystic teratoma, Mullerian cysts or epidermoid cysts, cystic change in solid neoplasms as in paraganglioma, neurogenic tumor or pseudomixoma retroperitonei and some non-neoplastic lesions such as pancreatic pseudocysts, lymphocele, urinoma and hematoma.

**Figure 2:**
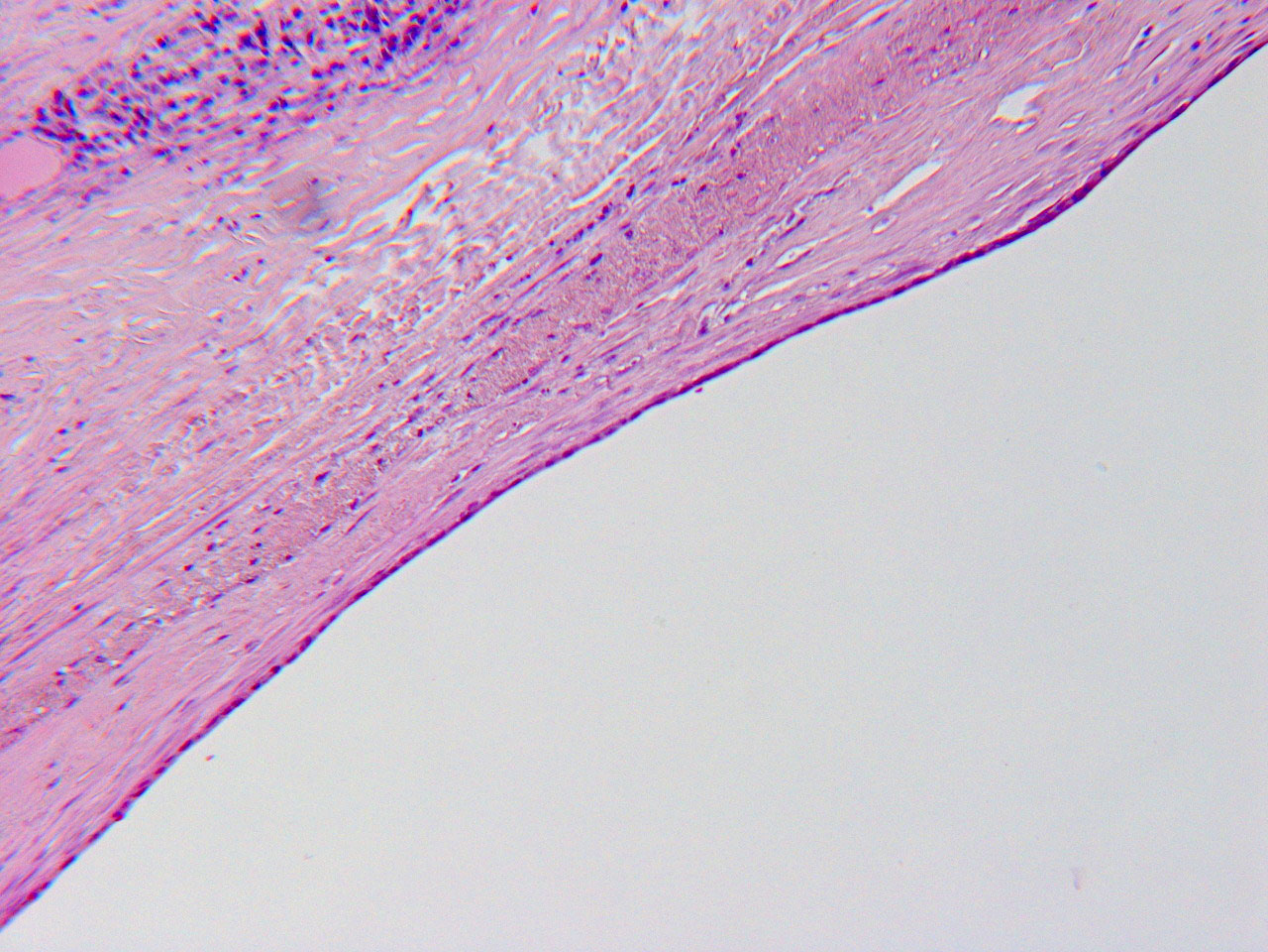
**A the microscopic view, it can be seen the cuboidal epithelium, sometimes cylindrical, with mucus-secreting cells (HE, 10×)**.

**Figure 3:**
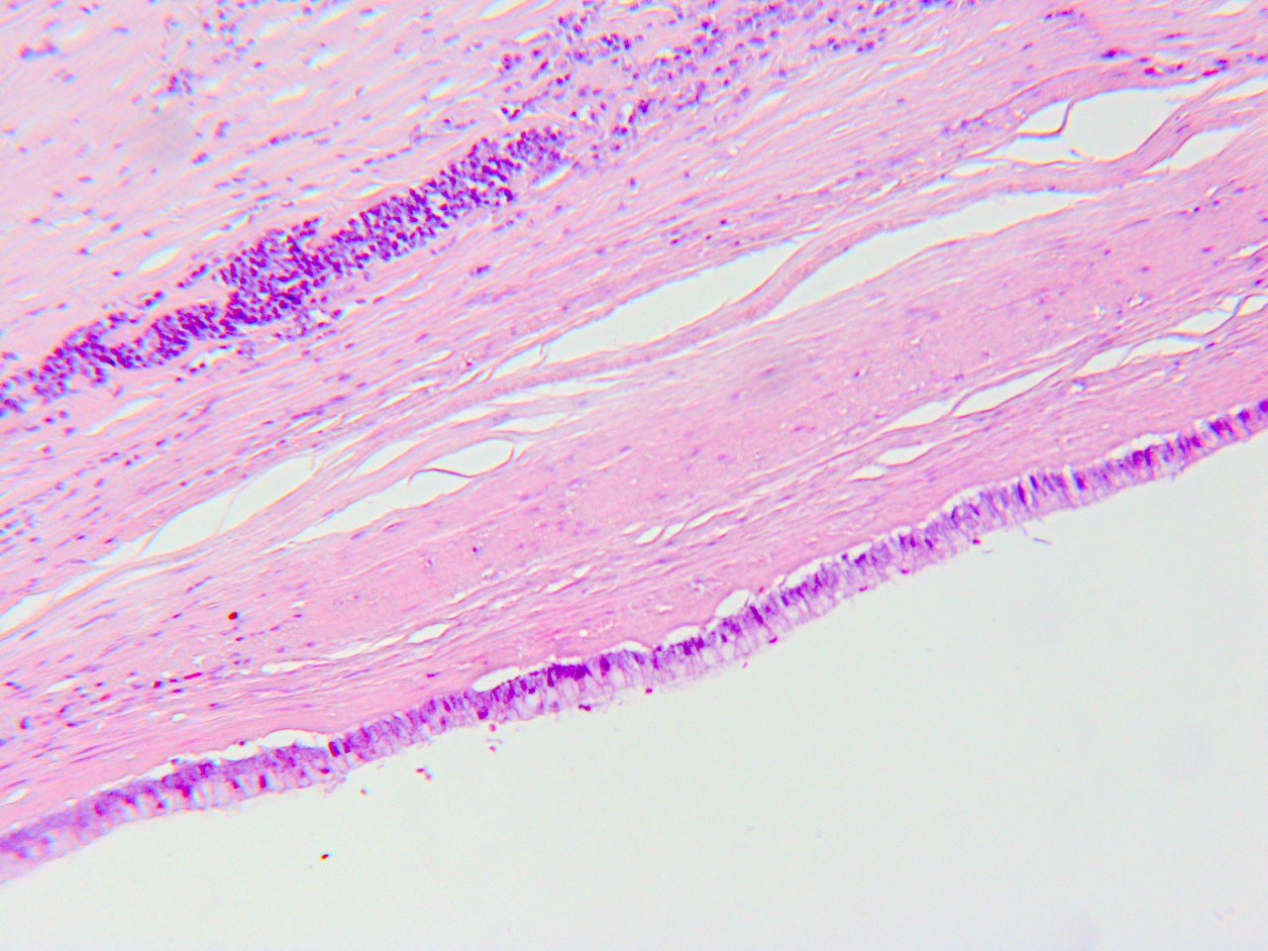
**Another microscopic view of the epithelium (HE, 20×)**.

**Figure 4:**
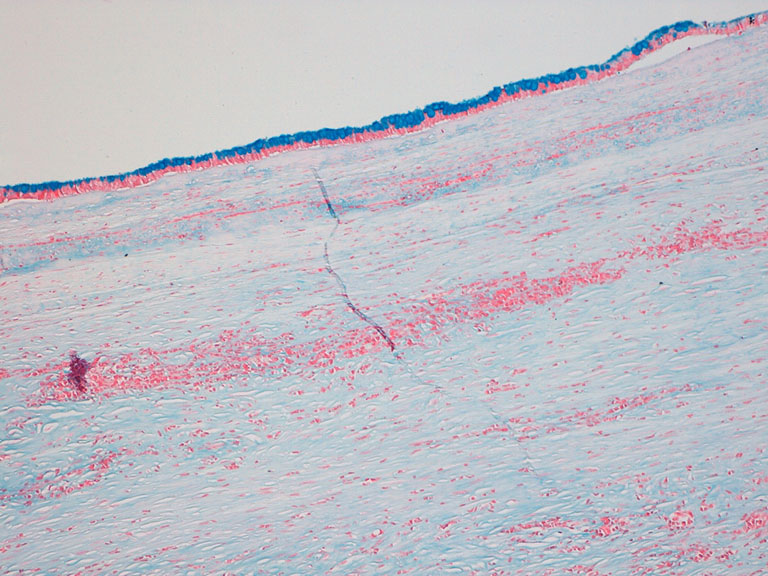
**Alcian blue staining identifies the mucinous secretion of the surface epithelium (10×)**.

Under histological examination the cyst wall was composed of a tick layer of connective fibrous tissue covered by a single-layer of cuboidal cells with interspersed typical goblet cells (fig. 2 and 3); ovarian-like mesenchymal stroma was not seen. We performed Alcian blue staining in order to highlight the mucinous secretion of the surface epithelial cells (fig. 4).

**Figure 5:**
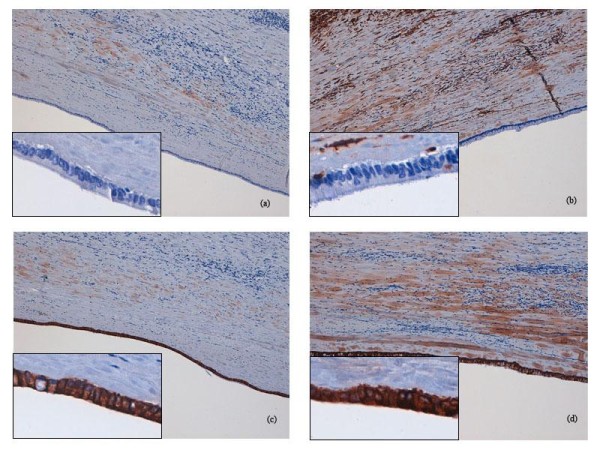
**Immunoreactivity for calretinin and vimentin is negative (a and b); cytokeratin 7 and 20 are positive (c and d) (10×, inset 40×)**.

Immunoistochemistry included cytokeratins 7 (DAKO; clone OV-TL 12/30; 1:30) and 20 (DAKO; clone Ks20.8, 1:50), which both identified the lining epithelium. Calretinin (DAKO; clone DAK-Calret 1, 1:50), a marker for mesothelial cells lineage was negative as well as vimentin (DAKO; clone V9, 1:50), (fig. 5a-d).

Because of the morphological and immunoistochemical features we made diagnosis of mesenteric mucinous cystoadenoma.

**Figure 6:**
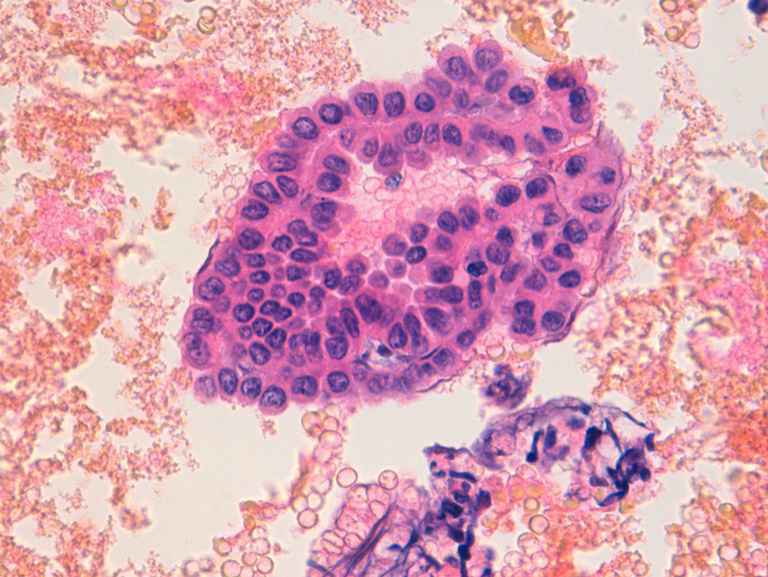
**Peritoneal washing shows red blood cells and reactive mesothelial cells (Cytoinclusion, HE, 63×)**.

The cytological examination of peritoneal washing fluid showed reactive mesothelial cells, and it resulted negative for the presence of malignant tumor cells. (fig. 6).

The patient was discharged after ten days, without complications. Two years after surgery, the patient is still in good health.

There are two theories about the genesis of retroperitoneal mucinous cystoadenoma: for its similarity with the ovarian cystoadenoma, it can originate from ectopic ovarian tissue [6].

The second hypothesis sustains that these cysts derive from the invagination of pluripotent embrionary epithelium and subsequent mucinous metaplasia, which would give origin to a mucinous cyst, and later, to the mucinous cystoadenoma (*coelomatic hypotesis*) [2]. According to this hypothesis, during the 4° week of pregnancy the embryo runs into complex processes of folding that will bring it to its three-dimensional form, with the constitution of the body cavities, organs and apparatus. We suppose that during these processes some cells (that will constitute the ovarian epithelium) can migrate in the serous cavity that will become the peritoneal one and, if adequately stimulated, these cells could undergo into mucinous metaplasia, with characteristics of benignity.

This case is similar to the one reported by Chen et al [1] regarding the age of the patient and symptoms, but it differs for the histology (the previous case has borderline malignancy) and for the surgical management (in the first case, surgeons voided the cyst under ultrasonic guidance before removing the mass).

## Conclusions

This case is relevant for the site of occurrence: most cystoadenomas are located in the ovaries, and mesenteric localization is rare.

Also reported symptoms are peculiar: the patient did not complain of pain or other symptoms related to compression of the right ureter (which was embedded in the mass with the right ovarian blood vessels) or bowel, but just a vague feeling of abdominal heaviness.

In our view, preoperative abdominal/pelvic computed tomography would have been useful

## Competing interests

The authors declare that they have no competing interests.

## Authors' contributions

All authors read approved the manuscript. ADG and MB analysed, interpreted the patient's data and drafted the manuscript. SF revised the pathology data and supervised the case report.

All authors read and approved the final manuscript.

## Pre-publication history

The pre-publication history for this paper can be accessed here:

http://www.biomedcentral.com/1471-230X/10/105/prepub
